# Stimulation of the Replication of ICP0-Null Mutant Herpes Simplex Virus 1 and pp71-Deficient Human Cytomegalovirus by Epstein-Barr Virus Tegument Protein BNRF1

**DOI:** 10.1128/JVI.01224-16

**Published:** 2016-10-14

**Authors:** Yongxu Lu, Anne Orr, Roger D. Everett

**Affiliations:** MRC-University of Glasgow Centre for Virus Research, Glasgow, Scotland, United Kingdom; Oregon Health and Science University

## Abstract

It is now well established that several cellular proteins that are components of promyelocytic leukemia nuclear bodies (PML NBs, also known as ND10) have restrictive effects on herpesvirus infections that are countered by viral proteins that are either present in the virion particle or are expressed during the earliest stages of infection. For example, herpes simplex virus 1 (HSV-1) immediate early (IE) protein ICP0 overcomes the restrictive effects of PML-NB components PML, Sp100, hDaxx, and ATRX while human cytomegalovirus (HCMV) IE protein IE1 targets PML and Sp100, and its tegument protein pp71 targets hDaxx and ATRX. The functions of these viral regulatory proteins are in part interchangeable; thus, both IE1 and pp71 stimulate the replication of ICP0-null mutant HSV-1, while ICP0 increases plaque formation by pp71-deficient HCMV. Here, we extend these studies by examining proteins that are expressed by Epstein-Barr virus (EBV). We report that EBV tegument protein BNRF1, discovered by other investigators to target the hDaxx/ATRX complex, increases the replication of both ICP0-null mutant HSV-1 and pp71-deficient HCMV. In addition, EBV protein EBNA-LP, which targets Sp100, also augments ICP0-null mutant HSV-1 replication. The combination of these two EBV regulatory proteins had a greater effect than each one individually. These findings reinforce the concept that disruption of the functions of PML-NB proteins is important for efficient herpesvirus infections.

**IMPORTANCE** Whether a herpesvirus initiates a lytic infection in a host cell or establishes quiescence or latency is influenced by events that occur soon after the viral genome has entered the host cell nucleus. Certain cellular proteins respond in a restrictive manner to the invading pathogen's DNA, while viral functions are expressed that counteract the cell-mediated repression. One aspect of cellular restriction of herpesvirus infections is mediated by components of nuclear structures known as PML nuclear bodies (PML NBs), or ND10. Members of the alpha-, beta-, and gammaherpesvirus families all express proteins that interact with, degrade, or otherwise counteract the inhibitory effects of various PML NB components. Previous work has shown that there is the potential for a functional interchange between the viral proteins expressed by alpha- and betaherpesviruses, despite a lack of obvious sequence similarity. Here, this concept is extended to include a member of the gammaherpesviruses.

## INTRODUCTION

Studies over the past 2 decades performed in several laboratories have established that there are many connections between the replication of different human herpesvirus members and cellular structures known as promyelocytic leukemia nuclear bodies (PML NBs, also known as ND10) (reviewed in references [Bibr B1][Bibr B2][Bibr B5]). The genomes of members of the alpha-, beta-, and gammaherpesvirus families have all been observed in close association with the proteins that make up PML NBs (6–10), and these viruses typically express proteins that disrupt the functions of one or more PML NB components (see reviews cited above and references therein). As described in the above-cited works, it has been established that one function of PML NBs is to limit the replication of many different classes of virus and that the viral proteins that disturb PML NB functions overcome these restrictive effects.

If such effects are of general importance in regulating the efficiency of certain viral infections, it is possible that the activities of a protein of one virus that targets PML NBs may be replaced by those of another viral protein with analogous functions, even if the viral proteins in question share little or no obvious sequence similarity. Over the past few years this hypothesis has been tested in a variety of scenarios. For example, it was found that the functions of herpes simplex virus 1 (HSV-1) immediate early (IE) protein ICP0 could be at least partially replaced by members of the ICP0 family of proteins expressed by other alphaherpesviruses (11). ICP0 induces the degradation or disrupts the functions of several PML NB components, for example, PML, Sp100, hDaxx, and ATRX (12–16), each of which has been shown to have a role in restricting herpesvirus infections. Human cytomegalovirus (HCMV) proteins IE1 (which targets PML and Sp100 [15, 17–20]) and pp71 (which interacts with and can induce the degradation of hDaxx and disrupts the hDaxx/ATRX complex [21–26]) also improve the replication of ICP0-null mutant HSV-1, and the two HCMV proteins in combination were almost as effective as ICP0 itself in the cell type examined (22). Conversely, prior expression of ICP0, similar to that of IE1, stimulates wild-type (wt) and pp71 mutant HCMV plaque formation and IE gene expression of IE1 mutant HCMV (27). In some ways even more strikingly, the Gam1 protein of chicken adenovirus (which induces degradation of PML [28, 29]) also strongly stimulates ICP0-null mutant plaque formation and gene expression (29). Thus, there are now many examples of at least partial functional replacement of one viral protein that affects PML NB activities by another.

In this report, we describe experiments that test whether proteins expressed by Epstein-Barr virus (EBV), a member of the gammaherpesvirus family, can also improve replication of ICP0-null mutant HSV-1. We constructed cells that express the EBV tegument protein BNRF1 (which disrupts the hDaxx/ATRX complex and is the functional, albeit unrelated, homologue of HCMV pp71 [5, 30, 31]) or EBV nuclear antigen leader protein (EBNA-LP) (which targets Sp100 [32, 33]) and also cells that express these EBV proteins in combination. We found that EBNA-LP and, to a greater extent, BNRF1 allowed improved ICP0-null mutant HSV-1 plaque formation, while the combination of these two proteins had an even greater effect. Furthermore, we found that BNRF1 could improve gene expression during pp71 mutant HCMV infection almost as efficiently as pp71 itself. These observations further strengthen the concept of a functional interchange between herpesviral proteins that have related effects on PML NBs or their components, even in the absence of apparent sequence similarity.

## MATERIALS AND METHODS

### Viruses and cells.

HSV-1 wild type (wt) strain 17+ was used, from which the ICP0-null mutant *dl*1403 was derived (34). Virus *dl*1403/CMVlacZ is a derivative of *dl*1403 that contains the *lacZ* gene under the control of the HCMV promoter/enhancer inserted into the *tk* gene was a gift from Chris Preston. The HCMV pp71 deletion mutant ADsubUL82 (35) was kindly provided by Tom Shenk and was used in conjugation with the AD169 parental wt strain. Human fibroblast (HFT) cells expressing ICP0 were used for the growth of stocks of ADsubUL82 (27). HepaRG cells (36) and a derivative expressing the tetracycline repressor (TetR) linked to enhanced green fluorescent protein EGFP (HA-TetR cells) have been described previously (37) and were grown in William's medium E supplemented with 10% fetal bovine serum Gold (PAA Laboratories, Ltd.), 2 mM glutamine, 5 μg/ml insulin, and 500 nM hydrocortisone. U2OS and HEK-293T cells and human diploid fibroblasts immortalized by expression of human telomerase from a lentivirus vector (38) (HFT cells) (lentivirus and cells kindly provided by David Davido and Chris Boutell, respectively) were grown in Dulbecco's modified Eagles' medium supplemented with 10% fetal calf serum (FCS). BHK cells were grown in Glasgow modified Eagles' medium supplemented with 10% new born calf serum and 10% tryptose phosphate broth. All cell growth media were supplemented with 100 units/ml penicillin and 0.1 mg/ml streptomycin.

### Plasmids and lentiviral vectors.

Lentivirus vector plasmids expressing the tetracycline repressor linked to a nuclear localization signal (NLS) and enhanced green fluorescent protein (pLKOneo.EGFPnlsTetR) and IE1 or myc-tagged pp71 from a tetracycline-inducible promoter have been described previously (22, 37). Analogous vectors expressing FLAG-tagged BNRF1 (with blasticidin resistance) and EBNA-LP (with either G418 or puromycin resistance) with a FLAG tag at its N-terminal end (with four W repeats, derived from plasmid pSG5-LP [39]; kindly provided by Paul Ling) were constructed. For single inducible expression of these proteins, the BNRF1 vector and the puromycin resistance version of the EBNA-LP were used to transduce HA-TetR cells. Some experiments utilized human fibroblast-derived HFT-TetR cells (27) which were transduced with the blasticidin-resistant BNRF1 vector. For expression of EBNA-LP in HFT-derived cells, sequential transductions were performed with a polycistronic vector expressing puromycin resistance and the tetracycline repressor (and also enhanced yellow fluorescent protein [EYFP] in an inducible manner [kindly provided by Chris Boutell]) with the G418-resistant version of the EBNA-LP-expressing vector. These cells could then be further transduced with the BNRF1 vector, thus creating HFT-derived cells that express both BNRF1 and EBNA-LP in an inducible manner. Vectors that expressed myc-tagged Zta, Rta, or EBNA1 were also constructed for this study using PCR of the relevant open reading frames, which were inserted in place of the ICP0 open reading frame in plasmid pLDT-cICP0 (37). The EBNA1 sequence is a version lacking most of the Gly-Ala repeat (40).

### Lentivirus transductions and induction of protein expression.

Lentivirus transduction, selection of transduced cells, and maintenance of cell lines were as described previously (41). Selection during routine culture used puromycin at 500 ng/ml, G418 at 0.5 mg/ml, or blasticidin (Invitrogen) at 1 μg/ml or combinations thereof, as relevant. The antibiotics were omitted from cells seeded for and during experimentation. For induction of protein expression, cells were treated with medium containing doxycycline (BD Biosciences) at 100 ng/ml for various times, as indicated in the text, and throughout the duration of an experiment.

### Virus plaque assays.

For determination of relative plaque-forming efficiencies of ICP0-null mutant HSV-1, cells were seeded for plaque assays into 24-well dishes at 1 × 10^5^ cells per well and then infected the following day with appropriate sequential 3-fold dilutions of *dl*1403/CMVlacZ. After virus adsorption, the cells were overlaid with medium containing 1% human serum, and then plaques were detected by staining for β-galactosidase expression in the cells within a plaque 24 h later (42). Relative efficiencies of plaque formation were calculated by comparing the number of virus plaques in each cell line in wells infected with the same dilutions of virus. Ratios over a number of dilutions were calculated and averaged, and the results presented were derived from at least two dilutions from at least two independent experiments. This method gives a more realistic comparison of plaque formation efficiencies than headline apparent titers because the plaque formation efficiency of ICP0-null mutant HSV-1 varies in a nonlinear manner with respect to dilution.

For assay of HCMV plaque formation, HFT-based cells were seeded into 24-well dishes, treated or not with doxycycline the following day, and then infected with the relevant HCMV at appropriate multiplicities of infection (MOIs). At 3 h after virus adsorption, the virus inoculum was removed and replaced with fresh medium. Plaques were stained at 10 days after infection by immunological detection of UL44, which is abundantly expressed in the cells that constitute a plaque. Cell monolayers were fixed with formaldehyde and treated with NP-40 as for immunofluorescence staining, washed twice with phosphate-buffered saline (PBS) containing 0.1% Tween 20 (PBST), and then treated with PBST containing 5% dried milk for 30 min before being incubated for 2 h at room temperature with anti-UL44 monoclonal antibody. Following three washes with PBST and incubation with horseradish peroxidase-conjugated goat anti-mouse secondary antibody for 1 h, the monolayers were washed with PBST three times, and plaques were revealed by incubation with 0.2 ml of True Blue solution (50-7802; Insight Biotechnology) for 10 min.

### Western blot analysis.

Cells were seeded into 24-well dishes at 1 × 10^5^ cells per well. After the relevant experimental manipulations, the cells were washed twice with PBS before being harvested in SDS-PAGE loading buffer. Proteins were resolved on 7.5% SDS gels and then transferred to nitrocellulose membranes by Western blotting. The following antibodies were used: anti-IE1/2 mouse monoclonal antibody (MAb) E13 (Serotec), anti-IE1 MAb 1B12 (a gift from Tom Shenk), anti-myc tag MAb 9E10 (Santa Cruz), anti-tubulin MAb T4026 (Sigma-Aldrich), anti-FLAG tag MAb M2, anti-UL44 MAb ab6501 (Abcam) or MAb 10D8 (Santa Cruz), anti-pp28 MAb ab6502 (Abcam), anti-pp71 MAb 2H10-9 (a gift from Tom Shenk), and anti-Sp100 rabbit polyclonal antibody (rAb) SpGH (43). Antibodies to actin and HSV-1 proteins ICP4 (MAb 58S), UL42 (MAb Z1F11), and VP5 (MAb DM165) were used as described previously (44).

### Immunoprecipitation.

Cells were seeded into 90-mm plates at 4.5 × 10^6^ cells per plate and then treated as relevant with doxycycline for 24 h to induce protein expression. The cells were washed twice with phosphate-buffered saline on ice, and then 1.5 ml of lysis buffer (87787; Pierce) supplemented with complete protease inhibitor cocktail (Roche Diagnostics) was added directly onto the cells on the plate. After incubation on ice for 30 min, the plate was scraped with a syringe plunger, and the harvested lysates were centrifuged at 13,000 rpm for 30 min at 4°C. The supernatant was precleared by incubation with protein G-agarose beads (16-201; Millipore) for 30 min at 4°C and end-over-end mixing; the beads were then removed by centrifugation at 13,000 rpm for 10 min. Aliquots of the clarified supernatant (0.5 ml) were incubated at 4°C overnight, with end-over-end mixing, with 1 μg of antibodies as follows: nonspecific preimmune rabbit IgG, anti-ATRX rabbit polyclonal antibody (rAb) H-300 (Sigma), or anti-hDaxx rAb 07-471 (Upstate). Mixtures were then incubated with 30 μl of fresh protein G-agarose beads for 60 min at 4°C with continuous mixing. The beads were washed four times with a buffer containing 10 mM Tris, pH 7.6, 1.5 mM MgCl_2_, 300 mM NaCl, 5% glycerol, 0.2 mM EDTA, 0.1% NP-40, and protease inhibitors, and then the beads were pelleted and resuspended in 30 μl of gel loading mix. Proteins were separated using 6.75% SDS-polyacrylamide Tris-glycine gels. After samples were subjected to electroblotting overnight (50 mA at 4°C), ATRX was detected using MAb 39F (a gift from Richard Gibbons), and hDaxx was detected by MAb Daxx-01 (Santa Cruz Biotechnology).

### Immunofluorescence and confocal microscopy.

Cells on 13-mm-diameter glass coverslips were fixed and prepared for immunofluorescence as described previously (45). PML was detected using MAb 5E10 (46), Sp100 was detected with rAb SpGH, hDaxx was detected with rAb 07-471, and ATRX was detected with rAb H-300. Antibodies against the FLAG tag (MAb M2) and the myc tag (MAb 9E10) were also used. The secondary antibodies used were Alexa 555-conjugated goat anti-mouse IgG and Alexa 633-conjugated goat anti-rabbit IgG (Invitrogen). The samples were examined using a Zeiss LSM 710 confocal microscope, with 488-nm, 561-nm, and 633-nm laser lines, with each channel scanned separately under image capture conditions that eliminated channel overlap. The images were exported as TIF files, minimally adjusted using Photoshop, and then assembled into figures using Illustrator.

## RESULTS

### Inducible expression of BNRF1.

**FIG 1:**
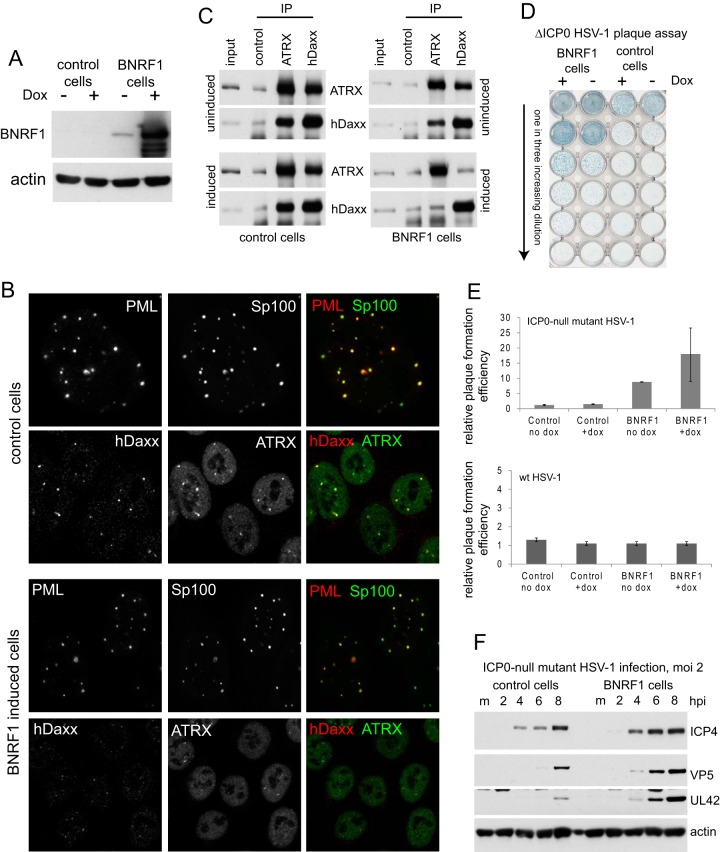
BNRF1 increases the replication efficiency of ICP0-null mutant HSV-1. (A) Inducible expression of BNRF1 in HepaRG-based cells after treatment with 100 ng/ml doxycycline (Dox) for 24 h. (B) Induction of BNRF1 expression causes the displacement of hDaxx and ATRX, but not Sp100, from PML NBs. Each row of three images shows the separated and merged channels for the relevant protein, as indicated. The top two rows show control HA-TetR cells; the lower pair show induced BNRF1-expressing cells. (C) Induction of BNRF1 expression causes the disruption of the hDaxx/ATRX complex. Extracts made from uninduced and induced control and BNRF1-expressing cells were used for immunoprecipitation (IP) with control, hDaxx, or ATRX antibodies, as marked. Substantial coimmunoprecipitation of hDaxx and ATRX occurred in the control extracts, but in BNRF1-expressing cells this was reduced to antibody control levels. (D) Expression of BNRF1 increases ICP0-null mutant plaque formation. Virus *dl*1403/CMVlacZ was used to infect uninduced and induced control and BNRF1-expressing cells at increasing 3-fold dilutions. Plaques were visualized by staining for the β-galactosidase marker gene at 24 h after infection. (E) Quantification of plaque formation efficiencies from replicate experiments of the type shown in panel D. Relative plaque-forming efficiency was calculated by comparing the number of plaques under the stated condition with that of the same input virus dilution. (F) Expression of BNRF1 increases the efficiency of viral gene expression in ICP0-null mutant HSV-1 infections. Induced control and BNRF1 cells were infected at an MOI of 2, and then samples taken at the indicated times after infection were analyzed for ICP4, VP5, and UL42 expression. hpi, hours postinfection.

**FIG 2:**
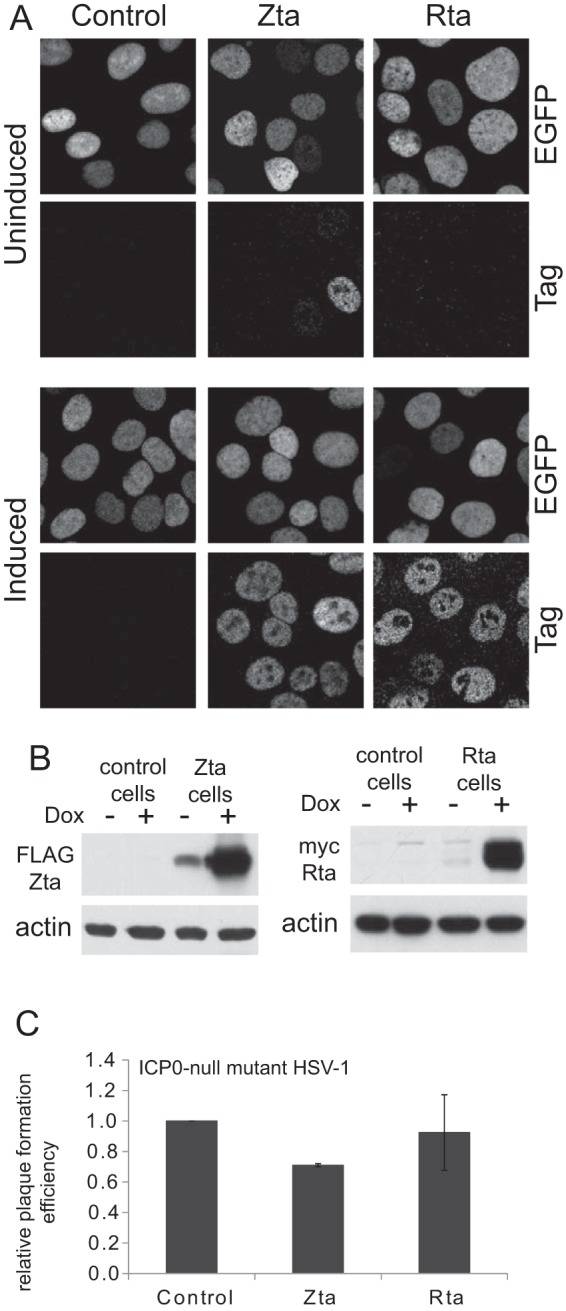
Expression of EBV proteins Zta and Rta has no effect on the efficiency of ICP0-null mutant HSV-1 infection. (A) HepaRG-based cells constructed to express FLAG-tagged Zta or myc-tagged Rta were analyzed by immunofluorescence before or after induction with doxycycline, as indicated. Pairs of images in vertical alignment show the EGFPnlsTetR fusion protein and the relevant epitope tag signals from the same field of cells. The controls are HA-TetR parental cells. (B) The same cells were analyzed by Western blotting for expression of FLAG-tagged Zta and myc-tagged Rta before and after induction. (C) ICP0-null mutant plaque formation was analyzed in induced control and Zta- and Rta-expressing cells. Dox, doxycycline.

Recent work has shown that BNRF1, a member of the ORF75c family of proteins that are represented in the teguments of many gamma herpesviruses (5), is required for efficient EBV infection through a mechanism involving interaction with hDaxx and disruption of the hDaxx/ATRX complex (30, 31). As these properties are analogous to the pp71 tegument protein of HCMV, which is, however, unrelated by sequence, and because pp71 can stimulate ICP0-null mutant HSV-1 plaque formation (22), we set out to investigate whether BNRF1 could also perform this function. A FLAG-tagged BNRF1 open reading frame was inserted in place of the ICP0 open reading frame of tetracycline-inducible lentiviral vector pLDT-cICP0 (37), and then HA-TetR cells (37) were transduced to isolate an HepaRG cell line allowing inducible expression of BNRF1. Analysis of these cells showed very abundant expression of BNRF1 following doxycycline treatment, with some expression detectable even before induction (Fig. 1A). Confirming previous data (31), we found that BNRF1 displaces hDaxx and ATRX from PML NBs without affecting PML or Sp100 (Fig. 1B) and disrupts the hDaxx/ATRX complex (Fig. 1C). We found that ICP0-null mutant HSV-1 had a pronounced increase in plaque formation in BNRF1-expressing cells, both before induction (presumably due to the leaky basal expression) and particularly after induction (Fig. 1D). Quantification of these increases gave about 10- and 20-fold effects, respectively, while BNRF1 expression had no effect on wt HSV-1 plaque formation efficiency (Fig. 1E). These increases were reflected in a general increase in IE, early, and late viral gene expression during ICP0-null mutant HSV-1 infection of BNRF1-expressing cells (Fig. 1F). These data are analogous to those previously observed in cells expressing HCMV tegument protein pp71 (22). As part of this study, we also studied the effects of the expression of EBV proteins Zta and Rta on ICP0-null mutant HSV-1 plaque formation. Despite successful expression of both proteins using analogous methods, we found no evidence that either protein affected ICP0-null mutant infection (Fig. 2).

### EBNA-LP targets Sp100 and augments ICP0-null mutant HSV-1 plaque formation.

**FIG 3:**
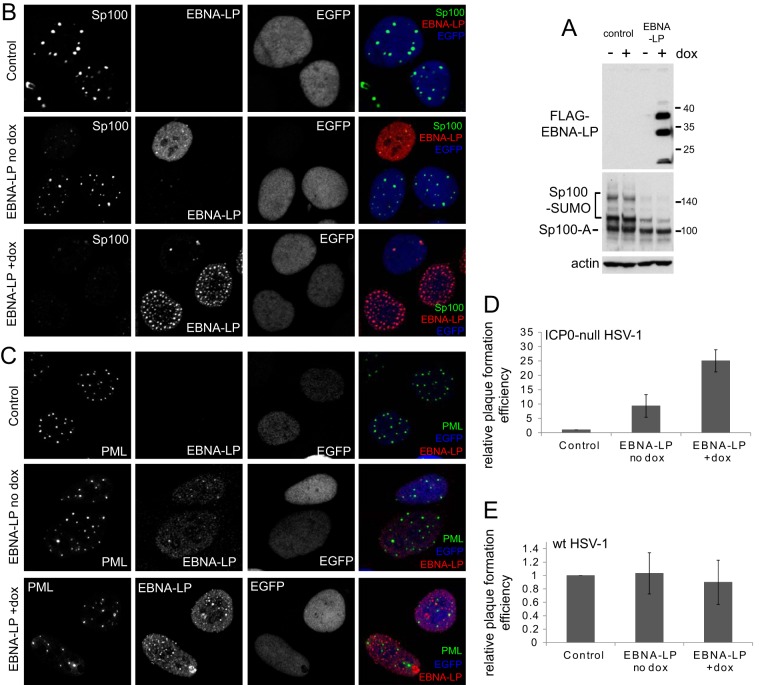
EBNA-LP increases the replication efficiency of ICP0-null mutant HSV-1. (A) Inducible expression of EBNA-LP in HepaRG-based cells after treatment with 100 ng/ml doxycycline (Dox) for 24 h. Analysis of Sp100 indicated substantial loss of the sumoylated forms of this protein in the BNRF1-expressing cells. (B) Induction of EBNA-LP expression causes the loss of Sp100 from PML NBs. Control, uninduced, and induced HFT EBNA-LP-expressing cells were stained for Sp100 and EBNA-LP as indicated. EGFP detects the nuclear localization of the EGFPnlsTetR fusion protein in these cells. A proportion of uninduced cells, particularly those expressing a low level of the TetR repressor, have detectable EBNA-LP expression, and in these cells Sp100 staining is much reduced. This phenotype is widespread in induced cells. (C) Cells as described in panel B were stained for PML and EBNA-LP. PML staining was unaffected by expression of EBNA-LP, whose punctate foci largely colocalized with PML in uninduced cells and to a lesser extent in induced cells. (D) Induction of EBNA-LP expression increases ICP0-null mutant plaque formation. Virus *dl*1403/CMVlacZ was used to infect uninduced control and EBNA-LP-expressing cells at increasing 3-fold dilutions, and then relative plaque-forming efficiencies were calculated by comparing the numbers of plaques in the two cell types with the same input virus dilutions. (E) EBNA-LP expression has no effect on the plaque formation efficiency of wt HSV-1.

The theme of this and other recent studies is that herpesviral proteins that target PML NB components may be partly interchangeable between different viruses. Another EBV protein that has been found to affect PML NBs is EBNA-LP, which displaces Sp100 from these structures (32, 33). Therefore, we constructed HA-TetR cells expressing FLAG-tagged EBNA-LP in an inducible manner. After induction, a variety of forms of EBNA-LP were expressed, with the slowest migrating major band having the expected mobility (Fig. 3A). Analysis of endogenous Sp100 in these cells showed that the sumoylated forms of the protein were greatly underrepresented compared to level in the parental cells (Fig. 3A, lower panel). This was also evident in the uninduced cells, likely because of significant leaky expression of EBNA-LP prior to induction (Fig. 3B and C), albeit at levels insufficient for detection on Western blots (Fig. 1A). The effect of EBNA-LP on the modified forms of Sp100 was not noted in the previous study (33), probably because the methods used here enable abundant expression of EBNA-LP in a very high proportion of the cells and also because of the effectiveness of the Sp100 antibody utilized. Induction of EBNA-LP expression caused widespread loss of punctate Sp100 staining, consistent with the previous data (33), and this also occurred in cells expressing EBNA-LP before induction (Fig. 3B). EBNA-LP had no apparent effect on the distribution of PML, however (Fig. 3C). ICP0-null mutant HSV-1 plaque formation increased by around 10- to 25-fold in uninduced and induced cells, respectively (Fig. 3D), without having any effect on wt HSV-1 (Fig. 3E). These results are consistent with a previous study that found that short hairpin RNA (shRNA)-mediated depletion of Sp100 enhanced ICP0-null mutant HSV-1 plaque formation (41).

### Analysis of human fibroblasts expressing selected EBV regulatory proteins.

**FIG 4:**
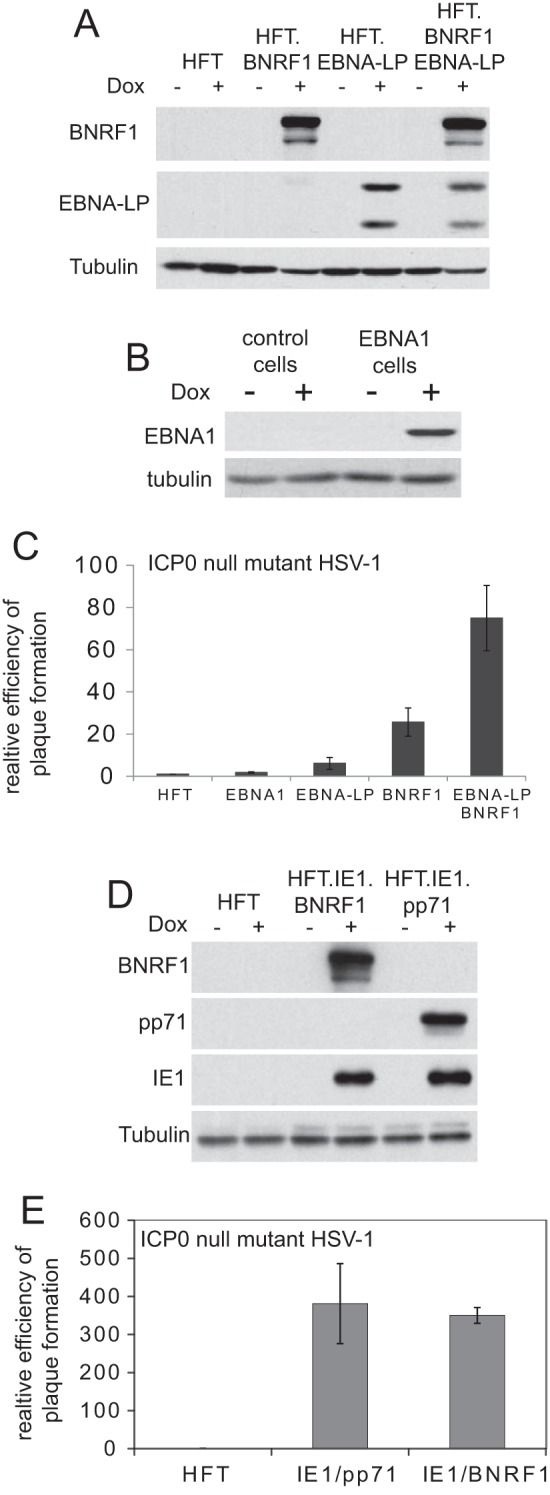
The effects of expression of BNRF1, EBNA-LP, and EBNA1 on ICP0-null mutant HSV-1 gene expression in HFT cells. (A) Western blot analysis of induced expression of EBNA-LP or BNRF1 or the combination of both in HFT-based cells. (B) Inducible expression of EBNA1 in HFT-derived cells, which were used for the control. (C) The efficiency of ICP0-null mutant HSV-1 plaque formation was analyzed in cells analogous to the above after treatment with doxycycline, using the approach described in the legends of Fig. 1 to 3. The results are expressed as fold increases above the level in control HFT cells. (D) Western blot analysis of cells constructed to express combinations of HCMV proteins IE1 and pp71 or of IE1 with BNRF1. (E) Plaque formation analysis on ICP0-null mutant HSV-1 in HFT-based cells expressing combinations of IE1 and pp71 or of IE1 and BNRF1 in comparison with control HFT cells.

In order to extend these analyses, we also constructed immortalized human fibroblasts (HFT cells) expressing EBNA1, BNRF1, or EBNA-LP. Western blot analyses of examples of these types of cells are shown in Fig. 4A and B (in this case showing only two of the forms of EBNA-LP being expressed). Immunofluorescence analysis of such cells gave results that were consistent with those in the HepRG-based cells described above although in the HFT cells EBNA-LP expression was detectable in only about 50% of cells. Both EBNA-LP and BNRF1 increased ICP0-null mutant plaque formation in these HFT cells, but EBNA1 had little effect (Fig. 4C). We have previously found that when HCMV proteins IE1 and pp71 are expressed in combination, they are able to increase ICP0-null mutant HSV-1 plaque formation to a much greater extent than use of either protein alone in both HepaRG and HFT cells (22, 27). In an analogous manner, we found that the combination of EBNA-LP and BNRF1 was considerably more effective than use of either alone (Fig. 4A and C) (see Materials and Methods for details of the construction of these cells).

We had previously found that prior expression of IE1 or the combination of IE1 and pp71 in HFT cells increased ICP0-null mutant plaque formation efficiency by about 14-fold and several hundred-fold, respectively, while pp71 by itself had a 9-fold effect (27). As a further test of the functional analogy between BNRF1 and pp71, we analyzed the effect of the combination of BNRF1 with IE1 in comparison to that in cells expressing IE1 and pp71 (Fig. 4D). Again, both combinations increased ICP0-null mutant plaque formation efficiency by several hundred-fold (Fig. 4E), which is far greater than the effect of IE1 alone (27) but still less than the greater than 2,000-fold increase obtained by prior expression of ICP0 (27).

### Inhibition of recruitment of PML NB proteins to HSV-1 genomes by EBV proteins.

**FIG 5:**
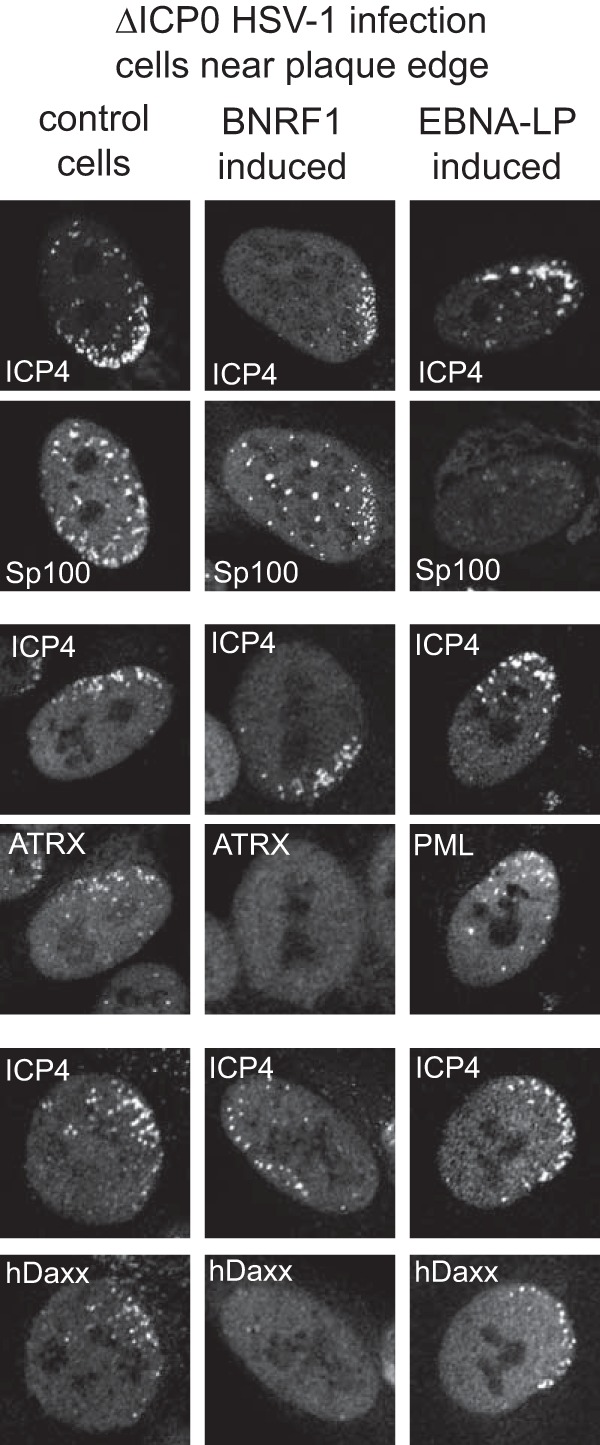
Effect of expression of BNRF1 and EBNA-LP on recruitment of PML NB proteins to sites associated with HSV-1 genomes. Control cells or cells induced to express BNRF1 (HepaRG-based) or EBNA-LP (HFT-based) were infected with ICP0-null mutant HSV-1 at a low multiplicity of infection, and then cells at the edges of developing plaques were analyzed for the localization of ICP4, Sp100, hDaxx, or ATRX as indicated. All of the PML-NB proteins were relocated to sites associated with ICP4 (which strongly binds to HSV-1 DNA) in control cells. Images show the signal of the indicated PML PB protein paired with that of ICP4. Recruitment of Sp100 but not hDaxx or ATRX occurs in BNRF1-expressing cells, while recruitment of PML and hDaxx but not of Sp100 occurs in cells expressing high levels of EBNA-LP.

A hallmark of PML NB proteins is their response in the early stages of infection that leads to their recruitment to sites that are closely associated with HSV-1 genomes (14, 45, 47). ICP0 inhibits this recruitment response, while IE1 and pp71 inhibit that of PML/Sp100 and hDaxx/ATRX, respectively, with these activities correlating with their stimulation of ICP0-null mutant HSV-1 infection (22, 27). We found that this principle extended to the EBV proteins under study in that BNRF1 inhibited recruitment of hDaxx and ATRX without affecting that of Sp100 (Fig. 5). In cells expressing abundant EBNA-LP in which the Sp100 signal was much reduced, the recruitment of Sp100 to ICP0-null mutant HSV-1 genomes was also greatly diminished (Fig. 5) although recruitment was still observed when Sp100 foci remained. Recruitment of PML and hDaxx appeared unaffected by expression of EBNA-LP (Fig. 5). These results are again consistent with the idea of some functional conservation, even in the absence of sequence similarity, of herpesvirus proteins that target PML NBs or their constituents. They are also consistent with the hypothesis that inhibition of recruitment correlates with relief from the repression mediated by this group of proteins.

### BNRF1 complements pp71 mutant HCMV.

**FIG 6:**
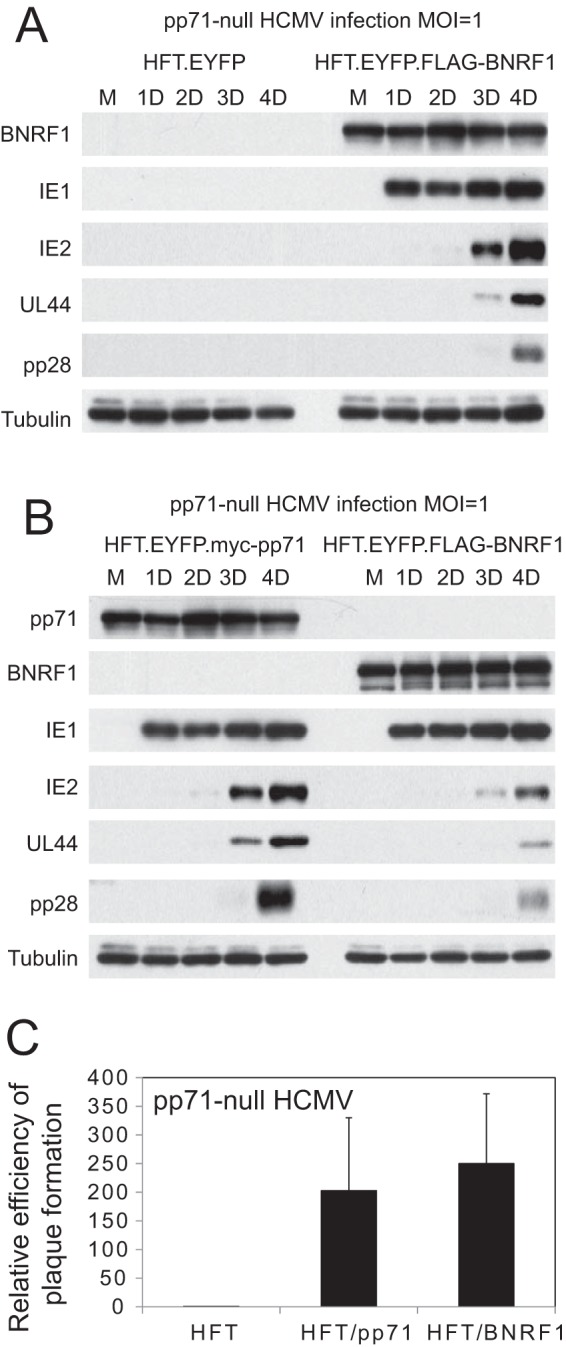
BNRF1 increases viral gene expression during pp71 mutant HCMV infection. (A) Control HFT cells expressing EYFP or FLAG-tagged BNRF1 were treated with doxycycline and then infected with AD169sub82 (MOI of 1). Samples were prepared over the following days and analyzed for expression of BNRF1 and HCMV proteins IE1, IE2, UL44, and pp28. (B) BNRF1 increases viral gene expression from pp71 mutant HCMV almost as efficiently as pp71. Cells induced to express myc-tagged pp71 or FLAG-tagged BNRF1 were infected with AD169sub82 (MOI of 1), and then samples were prepared over the following days and analyzed for expression of BNRF1 and HCMV proteins IE1, IE2, UL44, and pp28. (C) Plaque formation analysis of pp71-null mutant HCMV strain AD169sub82 in cells induced to express pp71 or BNRF1.

Given the functional analogies between BNRF1 and pp71, it was obviously of interest to determine whether BNRF1 could complement pp71-deficient HCMV. To this end, we utilized the HFT-derived cells that express BNRF1 in a similarly inducible manner (Fig. 4) and compared the results with those in cells that express pp71 using the same methodology (22). Infection of control HFT-derived cells with the pp71-null mutant HCMV strain ADsubUL82 illustrated that, as expected, very little HCMV gene expression was detectable while in induced BNRF1 cells viral IE, early, and late proteins were abundantly detected (Fig. 6A). Infection of pp71- and BNRF1-expressing cells in parallel with ADsubUL82 indicated that BNRF1 stimulated expression of IE1 to a similar extent as pp71 under these conditions, and while expression of IE2, UL44, and pp28 was not as efficient in BNRF1-expressing cells as in pp71-expressing cells, these proteins were still readily detectable (Fig. 6B). Furthermore, we found that expression of BNRF1 stimulated pp71-null mutant HCMV plaque formation to a degree similar to that of pp71 itself (Fig. 6C). These results conform to the expectation that BNRF1 is the functional orthologue of pp71 and are reminiscent of previous observations indicating a functional interchange between viral proteins that disrupt the hDaxx/ATRX complex (48, 49).

## DISCUSSION

The results presented here provide a further illustration that inducible expression cell lines constructed by the use of lentiviral vectors provide an effective approach to the study of viral protein function. We wished to extend previous analyses into the functional interchange between alpha- and betaherpesvirus proteins that affect PML NB components to the gamma-herpesviruses, based on previous studies from other groups. The results with BNRF1 are perhaps unsurprising, given the many established functional analogies between it and pp71 of HCMV (5, 31) and the fact that pp71 stimulates ICP0-null mutant HSV-1 plaque formation (22, 27). Here, we extend the functional similarities between the two proteins by showing that BNRF1 not only augments ICP0-null mutant HSV-1 replication but also stimulates pp71-deficient HCMV infection and gene expression with an efficiency similar to that of pp71 itself.

The results with EBNA-LP are consistent with the previous studies on its effects on Sp100 and PML NBs (32, 33) and on the increase in ICP0-null mutant plaque formation efficiency in Sp100-depleted cells (41). Here, we find that when EBNA-LP is expressed at low levels, it colocalizes with PML in PML NBs, and we confirm that high levels of EBNA-LP displace Sp100 from PML NBs, an effect that is probably connected with the loss of its sumoylated forms (Fig. 3A). On the basis of the observation that high-level expression of Sp100 led to activation of the EBNA2 promoter, it was previously concluded that the role of EBNA-LP was to release Sp100 from PML NBs so that it could act as a coactivator in concert with EBNA-LP (33). With the advent of RNA interference (RNAi)-mediated depletion methodologies, it became apparent that Sp100 acts as a repressor rather than an activator in several herpesvirus infections (18, 41, 50, 51), and the activation seen in earlier studies may be explained by dominant negative effects due to overexpression.

We have previously suggested that intrinsic resistance conferred by PML NBs has a modular aspect such that the repressive effects of PML, Sp100, and hDaxx/ATRX are additive (52). Thus, while ICP0 overcomes the effects of all of these proteins, HCMV IE1 or pp71 targets PML/Sp100 or hDaxx/ATRX, respectively, and the combination of the two HCMV proteins has almost as great an effect as that of ICP0 itself in HepaRG-based cells (22). We found further evidence that is consistent with this hypothesis during the current studies in that the combination of IE1 and BNRF1 stimulated ICP0-null mutant plaque formation to a similar extent as the IE1/pp71 combination (Fig. 4D). It is interesting that, throughout our series of studies on RNAi-mediated depletion of single or multiple PML NB components or expression of heterologous viral regulatory proteins, we have never been able to create cells which, like U2OS cells, completely fail to restrict ICP0-null mutant HSV-1. Accordingly, we have been careful never to state that PML NBs are the sole regulatory mechanism involved; indeed, it is known that other proteins and pathways also contribute to restriction. We would not expect any individual shRNA to substitute completely for ICP0, not only for the above reasons but also because we cannot exclude remaining trace expression, which could be too low perhaps for detection but nonetheless sufficient to retain some biological activity. Expression of heterologous viral proteins tends to have more marked effects than that of single shRNAs, maybe because these proteins achieve a greater inactivation of the restriction factor in question, but nonetheless they differ from ICP0 in that the latter seems uniquely capable of inactivating all the factors involved in restriction as a whole. Thus, a picture emerges of the full effect of restriction being made up of several separable components, with the suggestion that sequential inactivation of two or more such components leads to greater levels of relief from restriction. The most effective combinations found so far are IE1/pp71 and IE1/BNRF1, which would be expected to inactivate PML, Sp100, hDaxx, and ATRX. These combinations result in the restoration of ICP0-null mutant plaque formation efficiency to within 2-fold of the wt level in HepaRG cells or about 4-fold in HFT-based cells. Given the normal defect of several hundred- to a few thousand-fold, these degrees of complementation are very substantial.

Finally, the results reported here are consistent with the hypothesis that viral activators can be grouped into two broad types, those that function through virus-specific mechanisms (such as specific sequences in the viral genome) and those that impede cell-mediated restriction. Members of the former type would be expected to have virus-specific activities, while those of the latter may be in part functionally interchangeable between different viruses.
